# Supplementary material for: Comparative analysis of the lung microbiota in patients with lung cancer, chronic obstructive pulmonary disease, and community-acquired pneumonia
Source: Microbiol Spectr. 2026 Mar 16;14(4):e00450-25. doi: 10.1128/spectrum.00450-25 (PMC13055351; doi:10.1128/spectrum.00450-25)
Supplement: Supplemental figures and tables — Figures S1 to S4, and Tables S1 and S2. [file spectrum.00450-25-s0001.pdf]

# Supplementary materials

Comparative analysis of the lung microbiota in patients with lung cancer, chronic obstructive pulmonary disease and community-acquired pneumonia

Jia Xu[a,b,e#], Yingmiao Zhang[a,c#], Lifeng Shi[a], Hui Wang[a], Ming Zeng[d], and Zhongxin Lu[a,b,c†]

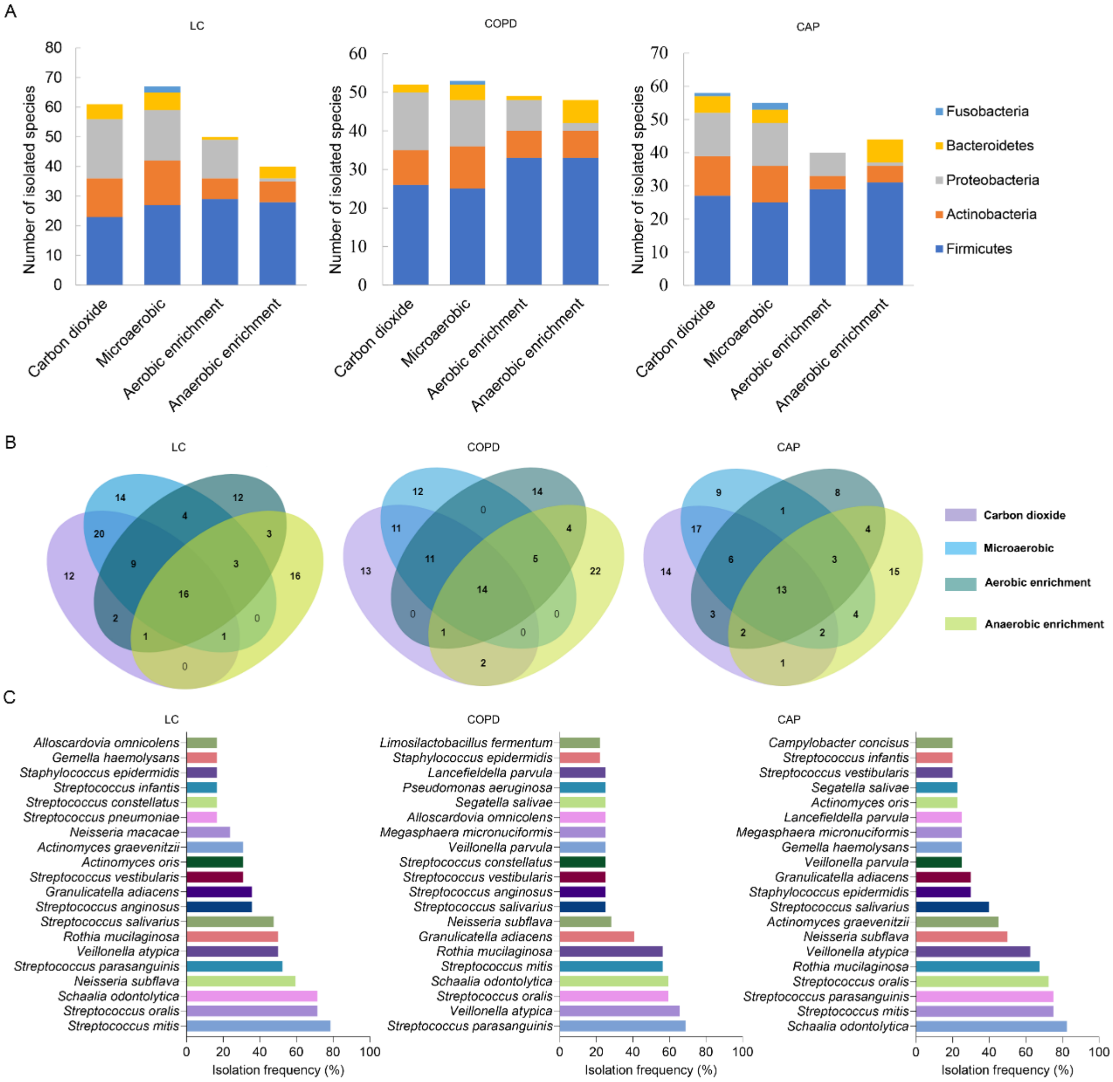


**Supplementary Figure S1** Characteristics of isolated bacteria across the three groups

under different culture conditions. (A) The distribution of bacteria isolated from each group of samples under different culture conditions at the phylum level. (B) Venn diagram showing culturable bacterial species isolated under different conditions within the three groups. (C) The top 20 bacterial species with the highest isolation frequencies in each group.

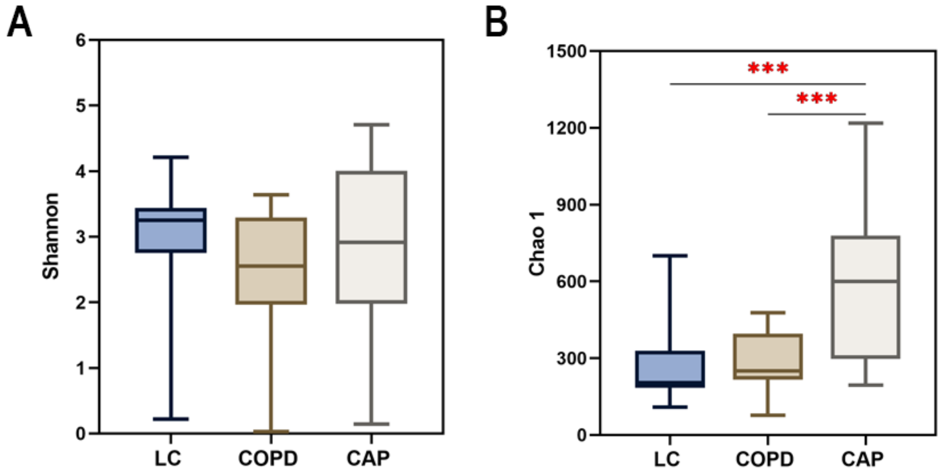


**Supplementary Figure S2** Analysis of microbial biodiversity in the lung microbiota ofpatients with LC, COPD, and CAP following rarefaction. The alpha diversity wasassessed using Shannon index (A), and Chaol index (B). ***, P<0.001. **, P<0.01.

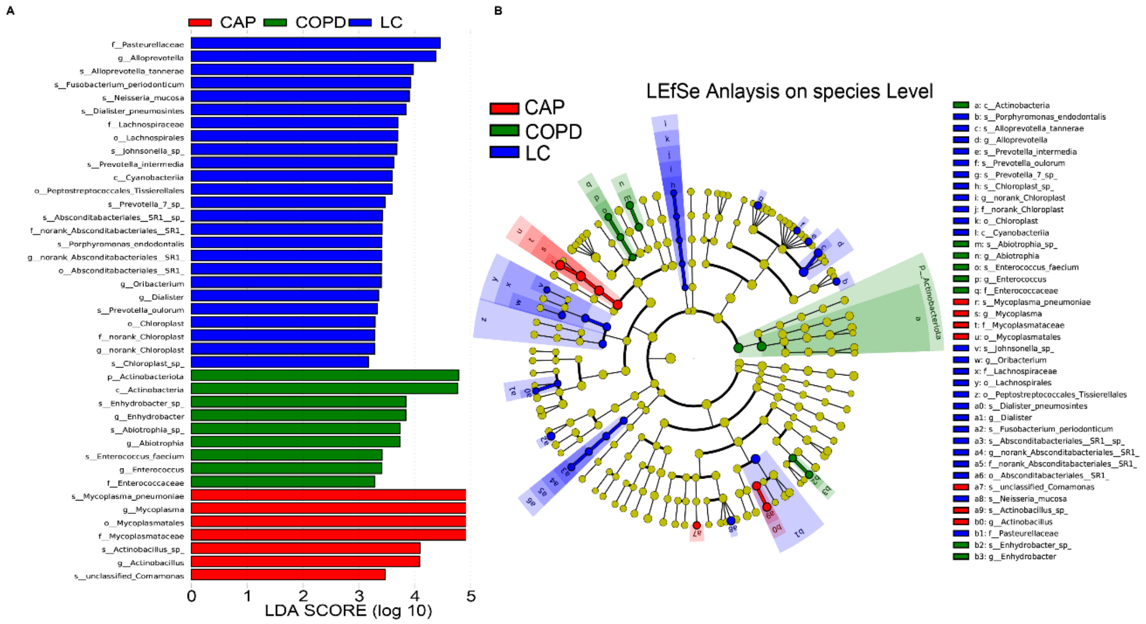


**Supplementary Figure S3** Taxonomic differences in lung microbiota among patients with LC, COPD, and CAP. (A) The linear discriminant analysis (LDA) scores of taxa presented the differences in microbiome composition among the three groups. (B) The taxonomic cladogram generated by Linear Discriminant Analysis Effect Size (LEfSe) analysis on species level showed the changes of microbiome in patients with LC, COPD, and CAP.

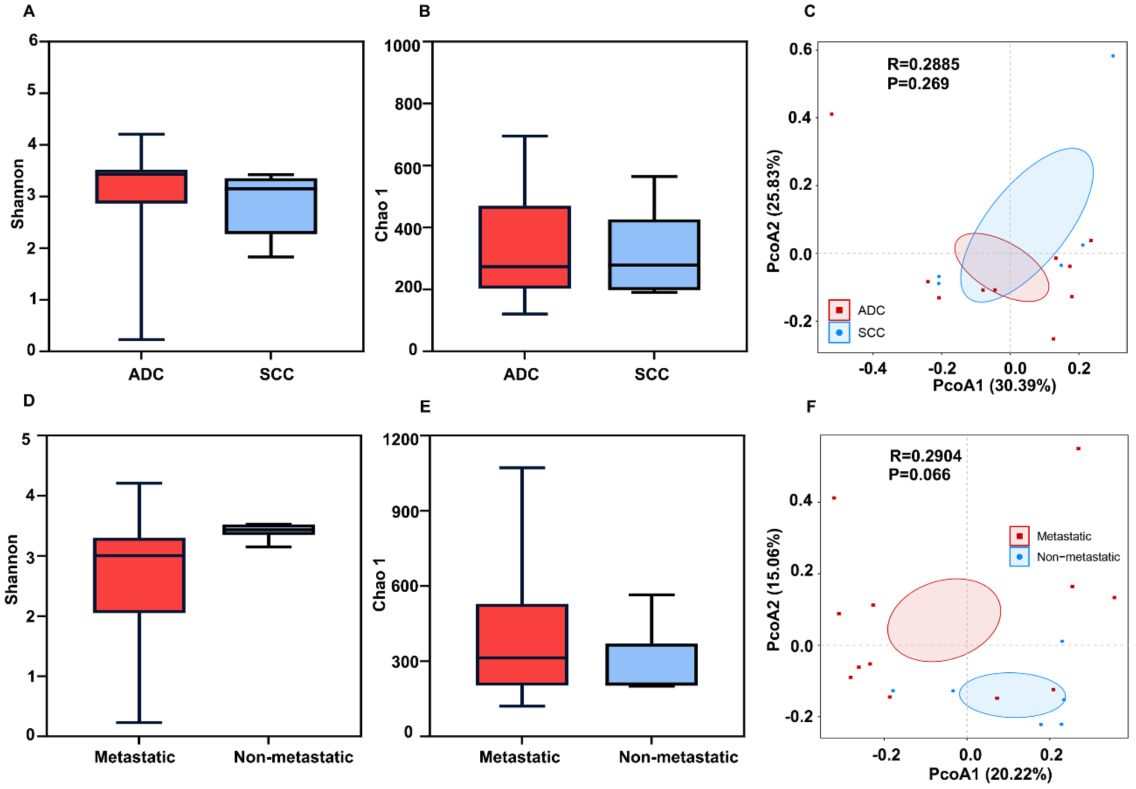


**Supplementary Figure S4** Diversity analysis of lung microbiotas in different subtypes of lung cancer.

(A-B) The alpha diversity was assessed using the Shannon index (P = 0.724) (A) and Chao1 index (P = 0.781) (B). (C) The Beta diversity was analyzed using Bray–Curtis distances (R = 0.2885, P = 0.269), with red and blue dots representing the Adenocarcinoma (ADC) and Squamous Cell Carcinoma (SCC) groups, respectively. (D-E) The alpha diversity was assessed using the Shannon index (P = 0.130) (D) and Chao1 index (P = 0.349) (E). (F) The Beta diversity was analyzed using Bray–Curtis distances (R = 0.2904, P = 0.066), with red and blue dots representing metastatic and non-metastatic groups, respectively.

**Supplementary Table S1** Number of bacterial species isolated from each sample under different culture conditions.

| Sample Number | Carbon dioxide | Microaerobic | Aerobic enrichment | Anaerobic enrichment | Total |
|---|---|---|---|---|---|
| LC01 | 3 | 7 | 3 | 4 | 10 |
| LC02 | 8 | 9 | 3 | 5 | 16 |
| LC03 | 8 | 8 | 5 | 5 | 16 |
| LC04 | 1 | 4 | 2 | 4 | 6 |
| LC05 | 3 | 8 | 2 | 3 | 9 |
| LC06 | 5 | 7 | 3 | 2 | 8 |
| LC07 | 7 | 10 | 4 | 3 | 15 |
| LC08 | 3 | 5 | 1 | 2 | 7 |
| LC09 | 5 | 8 | 1 | 3 | 13 |
| LC10 | 0 | 5 | 1 | 4 | 9 |
| LC11 | 9 | 11 | 5 | 5 | 22 |
| LC12 | 1 | 2 | 2 | 2 | 4 |
| LC13 | 7 | 8 | 6 | 3 | 15 |
| LC14 | 6 | 6 | 3 | 2 | 9 |
| LC15 | 6 | 9 | 0 | 3 | 12 |
| LC16 | 4 | 6 | 2 | 2 | 9 |
| LC17 | 5 | 5 | 2 | 5 | 14 |
| LC18 | 6 | 7 | 1 | 3 | 11 |
| LC19 | 5 | 6 | 2 | 2 | 9 |
| LC20 | 1 | 1 | 3 | 4 | 9 |
| LC21 | 8 | 5 | 1 | 3 | 14 |
| LC22 | 4 | 4 | 2 | 1 | 7 |
| LC23 | 2 | 4 | 4 | 7 | 13 |
| LC24 | 8 | 4 | 6 | 4 | 15 |
| LC25 | 6 | 11 | 0 | 6 | 17 |
| LC26 | 7 | 9 | 0 | 3 | 13 |
| LC27 | 10 | 8 | 2 | 6 | 20 |
| LC28 | 9 | 5 | 4 | 5 | 16 |
| LC29 | 6 | 7 | 3 | 4 | 14 |
| LC30 | 1 | 1 | 1 | 1 | 2 |
| LC31 | 8 | 9 | 4 | 2 | 14 |
| LC32 | 7 | 7 | 0 | 4 | 13 |
| LC33 | 9 | 8 | 5 | 6 | 21 |
| LC34 | 9 | 9 | 5 | 5 | 16 |
| LC35 | 9 | 5 | 1 | 3 | 12 |
| LC36 | 6 | 5 | 5 | 4 | 12 |

| | | | | | |
|---|---|---|---|---|---|
| LC37 | 6 | 6 | 3 | 4 | 16 |
| LC38 | 5 | 2 | 4 | 3 | 10 |
| LC39 | 5 | 6 | 5 | 8 | 16 |
| LC40 | 4 | 5 | 2 | 4 | 8 |
| LC41 | 7 | 10 | 4 | 2 | 15 |
| LC42 | 8 | 6 | 1 | 1 | 11 |
| COPD01 | 2 | 1 | 1 | 2 | 4 |
| COPD02 | 3 | 3 | 0 | 2 | 7 |
| COPD03 | 3 | 2 | 1 | 2 | 6 |
| COPD04 | 8 | 9 | 10 | 6 | 28 |
| COPD05 | 2 | 1 | 1 | 0 | 2 |
| COPD06 | 7 | 10 | 4 | 6 | 16 |
| COPD07 | 7 | 8 | 7 | 3 | 19 |
| COPD08 | 7 | 8 | 1 | 4 | 15 |
| COPD09 | 2 | 3 | 1 | 7 | 11 |
| COPD10 | 6 | 6 | 3 | 2 | 9 |
| COPD11 | 1 | 2 | 1 | 4 | 8 |
| COPD12 | 5 | 5 | 4 | 6 | 14 |
| COPD13 | 7 | 4 | 3 | 5 | 14 |
| COPD14 | 8 | 9 | 2 | 5 | 13 |
| COPD15 | 3 | 4 | 3 | 2 | 5 |
| COPD16 | 2 | 2 | 2 | 3 | 6 |
| COPD17 | 2 | 3 | 2 | 4 | 10 |
| COPD18 | 5 | 5 | 3 | 7 | 13 |
| COPD19 | 8 | 7 | 5 | 11 | 18 |
| COPD20 | 7 | 7 | 6 | 5 | 13 |
| COPD21 | 3 | 2 | 3 | 5 | 9 |
| COPD22 | 1 | 1 | 3 | 4 | 5 |
| COPD23 | 4 | 5 | 2 | 1 | 8 |
| COPD24 | 4 | 5 | 7 | 9 | 14 |
| COPD25 | 6 | 6 | 5 | 9 | 14 |
| COPD26 | 7 | 5 | 3 | 5 | 15 |
| COPD27 | 6 | 7 | 5 | 7 | 16 |
| COPD28 | 9 | 5 | 3 | 5 | 16 |
| COPD29 | 8 | 10 | 6 | 8 | 20 |
| COPD30 | 5 | 4 | 3 | 6 | 13 |
| COPD31 | 6 | 4 | 5 | 2 | 10 |
| COPD32 | 6 | 9 | 4 | 6 | 15 |
| CAP01 | 5 | 7 | 2 | 3 | 10 |
| CAP02 | 5 | 6 | 1 | 4 | 11 |

| | | | | | |
|---|---|---|---|---|---|
| CAP03 | 5 | 7 | 2 | 2 | 10 |
| CAP04 | 3 | 5 | 1 | 2 | 8 |
| CAP05 | 4 | 6 | 2 | 3 | 8 |
| CAP06 | 9 | 9 | 2 | 3 | 15 |
| CAP07 | 6 | 8 | 2 | 3 | 12 |
| CAP08 | 8 | 10 | 3 | 3 | 14 |
| CAP09 | 11 | 12 | 5 | 5 | 21 |
| CAP10 | 4 | 10 | 5 | 4 | 14 |
| CAP11 | 7 | 7 | 1 | 3 | 11 |
| CAP12 | 7 | 5 | 1 | 1 | 8 |
| CAP13 | 9 | 10 | 4 | 3 | 19 |
| CAP14 | 6 | 5 | 0 | 2 | 7 |
| CAP15 | 3 | 4 | 1 | 3 | 9 |
| CAP16 | 6 | 6 | 1 | 7 | 17 |
| CAP17 | 6 | 6 | 2 | 7 | 17 |
| CAP18 | 6 | 5 | 1 | 4 | 11 |
| CAP19 | 5 | 5 | 4 | 2 | 10 |
| CAP20 | 8 | 7 | 6 | 6 | 19 |
| CAP21 | 4 | 7 | 3 | 5 | 11 |
| CAP22 | 3 | 4 | 0 | 0 | 4 |
| CAP23 | 8 | 8 | 4 | 7 | 14 |
| CAP24 | 5 | 8 | 2 | 3 | 11 |
| CAP25 | 8 | 11 | 1 | 5 | 19 |
| CAP26 | 4 | 9 | 2 | 1 | 10 |
| CAP27 | 4 | 5 | 3 | 4 | 12 |
| CAP28 | 6 | 10 | 6 | 4 | 16 |
| CAP29 | 8 | 8 | 3 | 7 | 16 |
| CAP30 | 7 | 5 | 4 | 6 | 12 |
| CAP31 | 8 | 8 | 1 | 6 | 19 |
| CAP32 | 6 | 6 | 3 | 2 | 10 |
| CAP33 | 9 | 5 | 1 | 6 | 17 |
| CAP34 | 7 | 7 | 4 | 5 | 14 |
| CAP35 | 5 | 4 | 8 | 7 | 17 |
| CAP36 | 7 | 6 | 2 | 9 | 15 |
| CAP37 | 7 | 8 | 1 | 5 | 15 |
| CAP38 | 2 | 2 | 0 | 2 | 6 |
| CAP39 | 5 | 2 | 4 | 2 | 8 |
| CAP40 | 11 | 8 | 2 | 7 | 16 |

**Supplementary table S2** The basic information of bacteria isolated from clinical patients for the first time.

| No. | Original number | 16SrRNA length | Matched strains | Similarity (%) | First discovered location |
|---|---|---|---|---|---|
| 1 | COPD17A03 | 1400 | *Chryseobacterium siluri* | 99.64 | Liver of diseased catfish |
| 2 | CAP40B11 | 1413 | *Micrococcus antarcticus* | 99.58 | Antarctica |
| 3 | CAP33A04 | 1368 | *Sphingomonas hankookensis* | 98.68 | Wastewater |
| 4 | LC37B12 | 1380 | *Novosphingobium gossypii* | 99.42 | Gossypium hirsutum |
| 5 | LC36A04 | 1448 | *Radiobacillus deserti* | 99.45 | Desert soil |
